# Advances in research on immune escape mechanism of glioma

**DOI:** 10.1111/cns.14217

**Published:** 2023-04-23

**Authors:** Xu Guo, Gang Wang

**Affiliations:** ^1^ Department of Neurosurgery Cancer Hospital of Dalian University of Technology, Liaoning Cancer Hospital & Institute Shenyang Liaoning China; ^2^ Department of Neurosurgery the First Hospital of China Medical University Shenyang Liaoning China

**Keywords:** glioblastoma, glioma, immune escape, immunotherapy, review, tumor microenvironment (TME)

## Abstract

**Background:**

Glioma is the most common primary intracranial malignancy in clinical practice, and in particular, IDH‐wildtype glioblastoma has the worst prognosis. In recent years, surgical resection combined with simultaneous radiotherapy and immune checkpoint inhibitors has made some progress, but the efficacy is still not satisfactory, which may be related to the low immunogenicity of glioma cells and the tumor immunosuppressive microenvironment.

**Methods:**

A comprehensive review of relevant literature was conducted to explore the mechanisms by which tumors suppress antitumor immune responses and produce escape, with a focus on the immune cells in the tumor microenvironment (TME).

**Results:**

The mechanisms involved in immune evasion of glioma cells are complex and involve with immune cell differentiation and function.

**Conclusion:**

Our review emphasizes the need for a more profound comprehension of the mechanisms involved in immune response and immune evasion in glioma, to formulate more efficacious treatment modalities.

## Graphical abstract

Tumor cells escape immune recognition via downregulation of recognition the tumor cells, recruitment of immunosuppressive cells, production of immunosuppressive factors, activation of immune checkpoints, prevention of the infiltration of immune cells into the TME, etc. Here, we reviewed the current research on the mechanisms of immune response and immune escape of tumor cells in glioma particularly in IDH‐wild‐type glioblastoma.

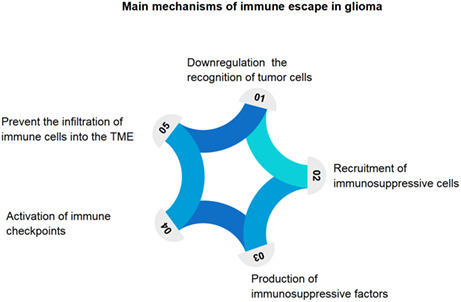

## 1 INTRODUCTION

As the most common primary intracranial malignant tumor in clinical practice, glioma is characterized by short survival period, high recurrence rate, high disability rate, and high mortality rate.^1^ Especially, the IDH‐wildtype glioblastoma is known as the most malignant, diffused, and aggressive.^2^ The main treatments for gliomas are surgery, radiotherapy, and chemotherapy.^3^ Surgery is the first step in the treatment of glioma. The boundary of high‐grade gliomas is unclear, it is difficult to completely remove the tumor tissue. For patients who progressed to high‐grade gliomas like glioblastomas, surgery is often accompanied by further radiotherapy, which includes local radiotherapy and stereotactic radiotherapy.^4^ Temozolomide is the most studied chemotherapeutic agent with clear efficacy in the treatment of glioma.^5^, ^6^ Also, the vascular targeting agent bevacizumab was found to be effective in recurrent high‐grade gliomas.^7^ Despite of this, the overall survival (OS) rate of gliomas has not been significantly improved. It is necessary and urgent to develop new treatments and approaches for glioma as a malignant tumor.

**FIGURE 1:**
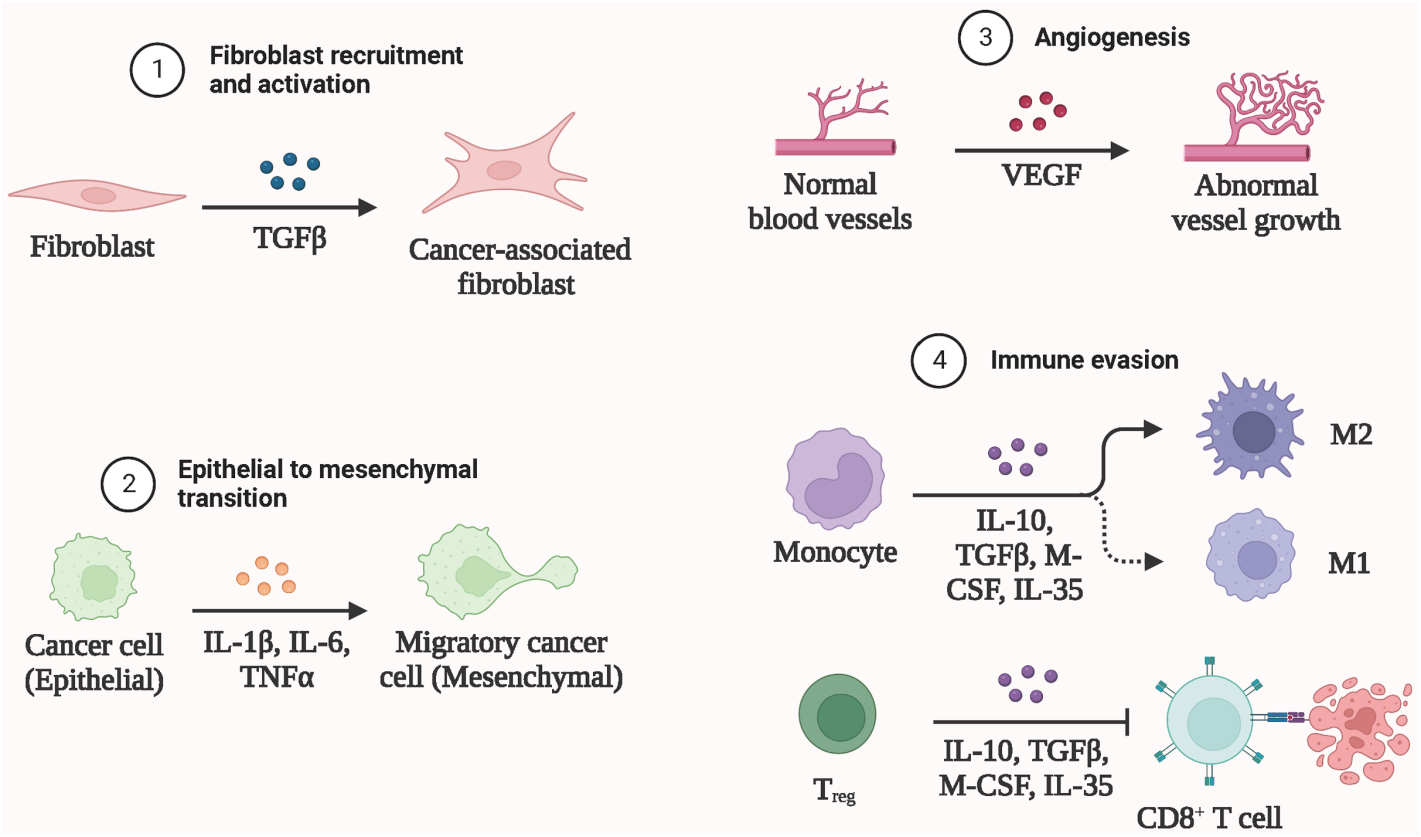
The biological processes in tumor microenvironment related to cancer malignant progression. The biological processes related to cancer malignant progression in the tumor microenvironment mainly include fibroblast recruitment and activation, epithelial mesenchymal transition, angiogenesis and immune evasion. Created with BioRender.com.

**FIGURE 2:**
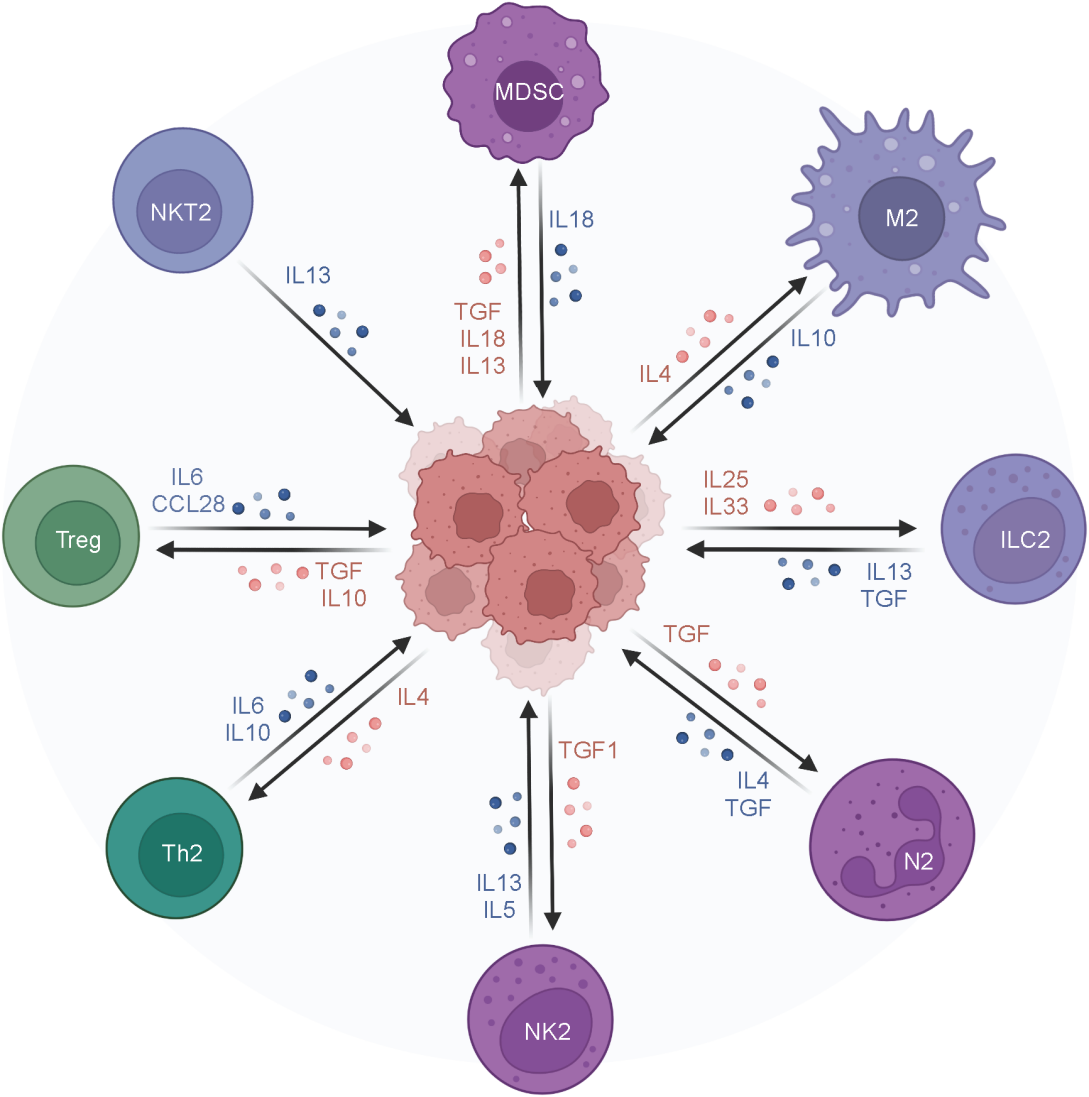
Immune cells involved in tumor immunosuppression. Many immune cells can participate in the process of tumor immunosuppression by interacting with tumor cells. These cells mainly include MDSC, M2, ILC2, N2, NK2, Th2, Treg, and NKT2. Created with BioRender.com.

Tumor microenvironment refers to the occurrence, growth, and metastasis of tumors, which are closely related to the internal and external environment of tumor cells, including not only the organizational structure, function, and metabolism of tumors but also the internal environment of tumors themselves. Tumor is closely related to its surroundings and its own environment, both of which are interdependent and antagonistic. Tumor cells can improve the conditions of development by self‐secretion and promoting paracrine secretion. The whole body and local tissues can also be changed by means of immunity, secretion, and metabolism to limit and affect the development of tumors, which is a very complex process. The biological processes in tumor microenvironment related to cancer malignant progression was shown in Figure 1. Immunotherapy is currently the most active and prospective research direction in the domain of tumor treatment. Immunotherapy is to stimulate the body's immune cells to respond to tumors by enhancing the function of immune effector cells, in order to achieve the purpose of removing tumor cells.^8^ Years of basic research on tumor immunity have been accumulated, and a mass of tumor immunotherapy has recently entered the clinical trial stage. Tumor immune escape stated for the phenomenon that tumor cells evade the recognition and attack of immune cells and immune molecules through various mechanisms, so that they can continue to grow and metastasize in the organism.^9^ Whereas, some malignant tumor cells will escape from the immune system and flee from the immune system.^10^ Therefore, the occurrence of immune escape by tumor cells is a vital strategy for tumor survival and progression. Numerous factors can contribute to the phenomenon of immune escape in tumor cells. These include mechanisms such as the shielding of tumor‐specific antigens, the removal of targets recognized by the body's immune system, a decrease in the population of immune cells capable of effectively killing tumor cells, an increase in immunosuppressive cells that facilitate tumor immune evasion, and an upregulation of immunosuppressive factors within the tumor microenvironment (TME).^11^ Immunosuppression is a key aspect of the mechanism by which tumor cells evade immune surveillance. One such mechanism involves the secretion of immunosuppressive cytokines by tumor cells, which promote the differentiation and proliferation of regulatory T cells (Tregs) and myeloid‐derived suppressor cells (MDSCs), leading to the manifestation of potent immunosuppressive effects.^12^ Within the tumor microenvironment, myeloid‐derived suppressor cells (MDSCs) may differentiate into tumor‐associated macrophages (TAMs), or they may directly suppress the activity of cytotoxic T lymphocytes (CTLs) or induce T cell inactivation, thereby promoting the establishment of an immunosuppressive milieu.^13^ The immune cells involved in tumor immunosuppression was displayed in Figure 2.

Investigations into the existence of lymphoid structures within certain regions of the brain tissue challenge the conventional notion of the central nervous system (CNS) as a partially immunologically privileged site, indicating that these structures can effectively and promptly clear foreign antigens or senescent and mutated cells.^14^ The ability of glioma cells to elude the host's immune surveillance arises from their low immunogenicity, elevated secretion of immunosuppressive factors, absence of adhesion and costimulatory molecules, and heightened presence of immunosuppressive cells within the tumor microenvironment.^15^ In this manuscript, we go over the researches on the mechanisms of immune response and immune escape of tumor cells in glioma to provide a reference for exploring and developing more effective immunotherapeutic options.

## 2 THE OVERVIEW OF GLIOMA TUMOR IMMUNE MICROENVIRONMENT

The glioma microenvironment pertains to the intrinsic and extrinsic surroundings that are intricately linked to the onset, progression, and metastasis of glioma, encompassing the tissue's structure, function, and metabolism in which the tumor cells reside, as well as the inherent milieu of the glioma cells themselves, which includes both the nucleus and cytoplasm.^16^ Tumor cells need to consume large amounts of nutrients to meet their metabolic needs, and the plasticity of tumor metabolism allows them to better adapt to a depleted or changing nutritional environment, which in turn reshapes the tumor immune microenvironment.^17^ The tumor immune microenvironment is divided into antitumor immune microenvironment and pro‐tumor immune microenvironment.^18^ The glioma‐associated microglia in the tumor microenvironment, the pro‐tumor effects of macrophages (GAMs), Treg, and the inactivation of natural killer (NK) cells can all diminish the antitumor influences of the organism and are closely linked to the constitution of the glioma immunosuppressive microenvironment.^19^ Gliomas have a unique immune microenvironment infiltrated by two main types of immune cells, microglia, and tumor‐associated cells (TAM, mainly M2‐type tumor‐promoting macrophages).^20^ They not only play the role of phagocytosis of tumor cells but also perform antigen presentation and secrete some inflammatory factors. After tumorigenesis, the inhibitory microenvironment leads to a decrease in phagocytosis and antigen‐presenting functions of both cells and promotes TAMs to M2 polarization, among others.^21^ And both cells express PD‐L1 on their surface, and they both induce a decrease in T‐cell activity through the mechanism of immune check sites. They also express PD‐1 on their surface, which inhibits their phagocytosis and antigen‐presenting ability by binding to PD‐L1 ligands on tumor cells, etc.^22^ TILs are the main antitumor cells, but in glioma, the existence of the blood–brain barrier causes difficulties in the recruitment of T cells. Not only the number of TILs is low but also the presence of receptors such as PD‐1 and CTLA4 on TILs can inhibit their antitumor effects after binding to ligands such as PD‐L1 and CD86 on tumor cells.^23^ And nowadays, the application of immune checkpoint locus inhibitors can help restore the function of killer T cells within the tumor, which in turn can perform tumor‐killing function. Dendritic cells (DCs) are specialized antigen‐presenting cells that accomplish antigen presentation and activation of T cells mainly in the brain or deep cervical lymph nodes, but the immune microenvironment of glioma suppresses the function of DC cells.^24^ Also, there are two suppressor cells in glioma, Treg, and MDSC, which are closely related to PD‐L1 expression in tumor cells.^25^

Cells within the microhabitat of glioma stem cells (GSCs) secrete a variety of cytokines to stimulate glioma stem cell self‐renewal, induce angiogenesis, and recruit immune cells to promote tumor cell invasion and metastasis.^26^ Microhabitat is a specialized microenvironment that can be regulated by regulating tumor stem cells (TSCs) both in the form of direct intercellular contact and secretion of cytokines.^27^ Among tumor microenvironments (TME), microhabitat is an anatomically unique microenvironment. Glioma‐induced cells have high adaptability, high plasticity, strong self‐renewal capacity, unlimited proliferation capacity, and multidirectional differentiation potential, which are properties that allow them to survive under adverse conditions and suggest that the stem cell microenvironment is necessary to maintain stem cell properties, metabolic plasticity.^28^ There is a strong link between glioma stem cells and the microenvironment in which they live. In the stem cell microenvironment, glioma stem cells can induce differentiation of bone marrow‐derived monocytes and microglia into TAMs, the latter being the major inflammatory cell population in the TME.^29^ Multiple lines of evidence suggest that TAMs have a role in promoting tumorigenesis, growth, invasion, and metastasis, affecting tumor metabolism.^30^, ^31^ Microhabitat maintains the essential properties of glioma stem cells, maintaining their phenotypic plasticity and thus allowing them to effectively evade surveillance by the immune system.^32^

Recently single‐cell analysis has emerged as a powerful tool to study the heterogeneity of gliomas at the single‐cell level and to identify the cellular and molecular mechanisms involved in immune escape.^33^ Single‐cell RNA sequencing (scRNA‐seq) has been used to identify different subpopulations of glioma cells and to characterize their gene expression profiles.^34^ This has led to the identification of novel immune checkpoint molecules and immunosuppressive pathways that contribute to glioma immune escape.^35^, ^36^ In addition, single‐cell analysis has also been used to study the immune microenvironment of gliomas, including the infiltration of immune cells and their functional states.^35^ Furthermore, single‐cell analysis has provided insights into the dynamic changes in the immune microenvironment during glioma progression and in response to immunotherapy.^37^ Overall, single‐cell analysis has significantly advanced our understanding of the complex immune escape mechanisms in gliomas and has provided potential targets for novel immunotherapeutic strategies.

## 3 CHARACTERISTICS OF THE COMPONENTS OF THE GLIOMA IMMUNE MICROENVIRONMENT INVOLVED IN THE IMMUNE ESCAPE MECHANISM

**FIGURE 3:**
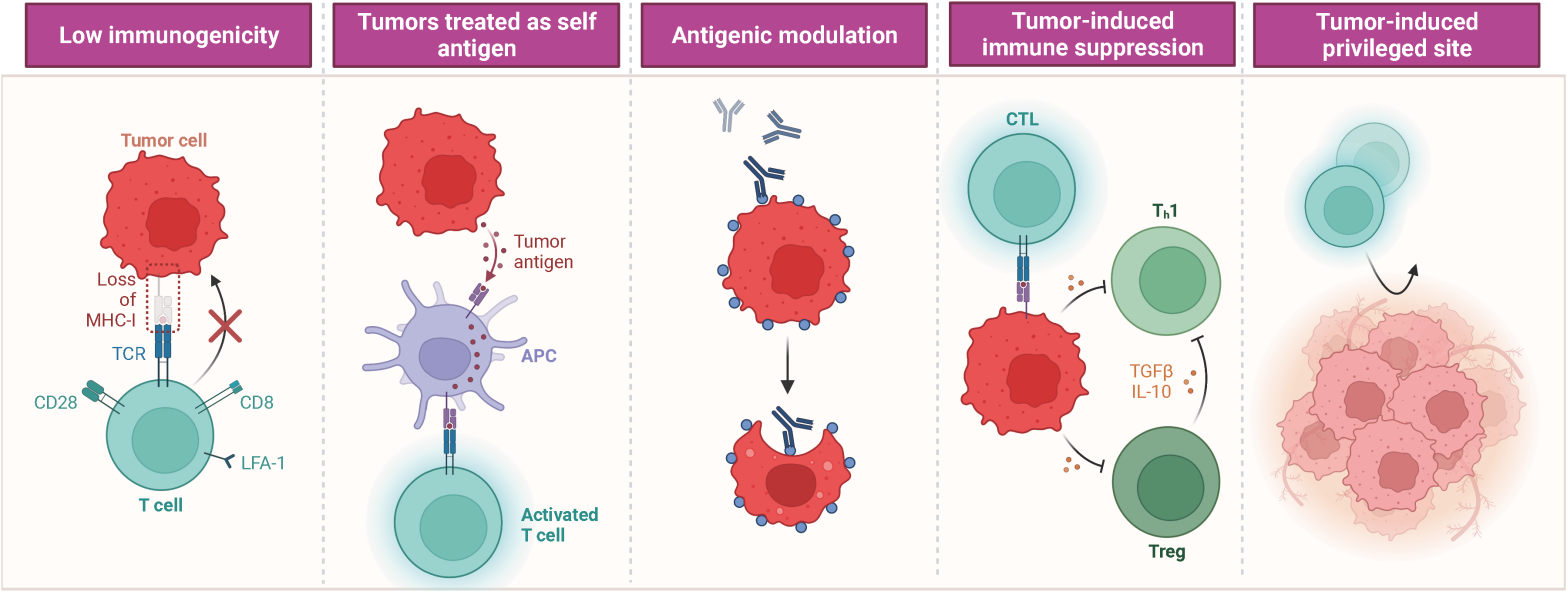
The ways of tumor cells escaping immune recognition. Tumor cells can escape immune recognition in many ways to achieve malignant growth and metastasis. Tumor cells escape immune recognition due to low immunogenicity, tumors treated as self antigen, antigenic modulation, tumor‐induced immune suppression and tumor‐induced privileges site. Created with BioRender.com.

The glioma immune microenvironment is composed of various immune cells and factors that interact with the tumor and play a critical role in tumor progression and response to therapy. Immune escape mechanisms are a significant challenge in glioma treatment, and the involvement of different immune components in these mechanisms has been extensively studied. (Figure 3).

### 3.1 Tumor‐infiltrating T lymphocytes and immune escape

**FIGURE 4:**
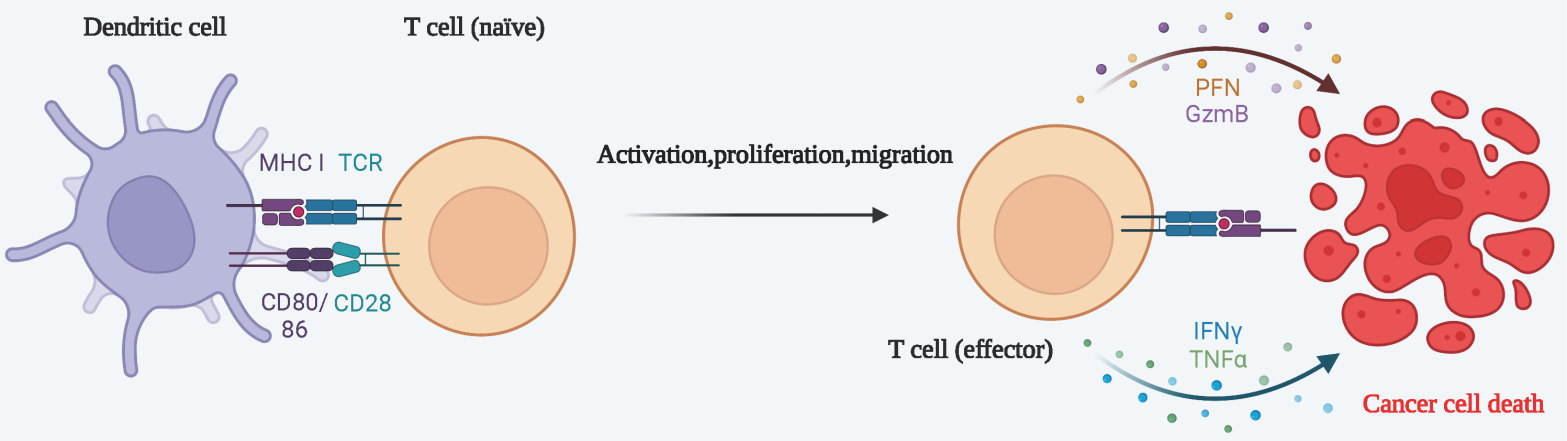
The process of T cell activation. After tumor cells release antigens, dendritic cells can present antigens captured on MHC class I molecules to T cells. When T cells encounter antigen‐presenting cells, they will start an activation process, which requires three sets of signals: molecules generated by TCR, costimulatory molecules and cytokines. After being stimulated by antigen, T cells proliferate, differentiate and transform into effector T cells. Activated T cells infiltrate the tumor microenvironment through blood vessels, and activated T cells recognize and kill targeted tumor cells. When the same antigen enters the cells of the body again, the direct killing effect of effector T cells on the antigen and the synergistic killing effect of cytokines released by effector T cells. Created with BioRender.com.

Tumor‐infiltrating lymphocytes (TILs) are a heterogeneous population of lymphocytes prevalent in tumor parenchyma and tumor mesenchyme, involved in tumor immune response and adjustment of cancer cell growth.^38^, ^39^ TILs include T cells, B cells, NK, and DCs. CD3 antigen is usually expressed on T lymphocytes and is an antigenic marker of T lymphocytes, mainly divided into CD3+ CD8+ T lymphocytes and CD3+ CD4+ T lymphocytes (helper T cells). Some memory T cells expressing CD29 and CD45RO.^40^ The subsets of helper T cells are divided into helper T cells 1 (Th1), helper T cells 2 (Th2), helper T cells 17 (Th17), and follicular helper T cells (Tfh), depending on the antigen and function.^41^, ^42^ CD8+ T lymphocytes are associated with a good prognosis^43^ and Treg cells are linked to a poor prognosis, but the specific immune effects also take into account the infiltration characteristics of the immune cells, that is, type, location, number, cell ratio, etc.^44^ The process of T cell activation was displayed in Figure 4.

Tumor‐infiltrating T lymphocytes also have an important role in the immune escape of glioma (Table 1). Studies have shown that miR‐21 expression is significantly increased in both glioma cells and tissues. Inhibition of miR‐21 inhibited the growth, migration, and invasion of glioma cells and accelerated apoptosis, and increased CD8+ T proliferation and cytotoxic activity. In addition, miR‐21 is also enriched in BMDM exosomes and can be delivered to glioma cells to promote immune escape by inhibiting PEG3 expression.^45^ Zhong et al. showed that SDC1 was closely connected with immune infiltration of gliomas in TME, especially activated CD4+ T cells and CD8+ T cells. sDC1 may regulate antigen processing and presentation in gliomas through CTSL or CD4, and thus participate in glioma immune escape.^46^ INPP4B expression was significantly reduced in gliomas, and overexpression of INPP4B suppressed glioma expression. Overexpression of INPP4B inhibited glioma cell proliferation, migration, apoptosis resistance, PD‐L1 expression, and T cell suppression. Mechanistic findings suggest that INPP4B inhibits immune escape from glioma by downregulating PI3K/AKT signaling and thereby suppressing PD‐L1 expression.^47^ The PD‐1 expression on tumor‐infiltrating T cells from glioma cell suspensions was examined and the circulating T cells was simultaneously isolated. The results showed a significant increase of PD‐1 in the TME compared to the blood circulation, but PD‐1 expression in the peripheral circulation of glioma patients was not different compared to normal controls. The results of animal experiments showed that the use of monoclonal PD‐1 blocking antibodies significantly increased the attainment of long‐term survival and prolonged survival time in mice. A significant rise in the proportion of CD8+ T cells and a significant decrease in the proportion of Tregs were detected after implantation of GL261 tumors in mice. These results suggest that PD‐1 blocking antibody may be a key target to inhibit glioma‐induced immune escape.^48^ The expression of endothelin B receptor (NTBR) was increased in both glioma tissues and cells, and the expression of NTBR was also closely associated with the clinical stage of malignant glioma.^49^ GARP was expressed in GBM tissues and cell lines as well as in low‐grade gliomas, and GARP was localized not only on the cell surface but also in the cytoplasmic and nuclear compartments of tumor cells. Mechanistic experiments showed that GARP can act as an immunomodulatory molecule by suppressing the function of e‐carrier T cells and thus promoting an immunosuppressive TME in primary brain tumors.^50^ It was shown that downregulation of MHC‐I and APM component expression was connected to activation of the Wnt pathway in glioma stem cells (GSCs), and inhibition of histone deacetylase (HDAC) significantly promoted the expression of MHC‐I and APM components, while activation of the Wnt pathway reversed this effect. The antitumor effect of tumor lysate vaccines could be significantly reduced by blocking CTL‐mediated killing. In conclusion, inhibition of the stem cell pathway may be an effective means of suppressing immune escape tumors in gliomas.^51^ Inducible T‐cell costimulatory ligand (ICOSLG) expression was found to be increased in GBM tissues and closely linked to poor prognosis in GBM patients by bioinformatics analysis and analysis of GBM tissue microarray assays. ICOSLG expression was increased in a TNF‐α/NF‐κB‐dependent manner in mesenchymal stem cells (MSCs) and promoted the expansion of interleukin‐10‐producing T cells. Inhibition of ICOSLG expression significantly inhibited GBM tumor growth and reduced interleukin‐10 levels in TME in mice.^52^ Du et al. found that Wnt ligand and activated EGFR induced the binding of β‐catenin/TCF/LEF complex to the CD274 gene promoter region and thus promoted PD‐L1 expression, in which AKT activation also played a significant role. Inhibition of β‐linked protein, AKT, or PTEN expression promotes PD‐L1 expression and enhances CD8+ T cell activation and tumor infiltration, and significantly restrain tumor growth and prolongs survival time in mice. Combination therapy with clinically available AKT inhibitors and anti‐PD‐1 antibodies overcame tumor immune evasion and greatly inhibited tumor growth.^53^ It showed that the expression levels of TLX and PD‐L1 were significantly and positively correlated with the macrophage‐mediated immunosuppressive phenotype in gliomas, while the expression levels of TLX were also positively correlated with the expression of PD‐L1. Inhibition of TLX expression significantly inhibited tumor growth and promoted antitumor immune responses by decreasing PD‐L1 expression, glioma‐associated macrophage numbers, and increasing cytotoxic lymphocyte infiltration. Mechanistic findings suggest that TLX directly binds to the promoter region of PD‐L1 and activates PD‐L1 transcription. In conclusion, TLX can lead to malignant progression and immunosuppression of glioma through transcriptional activation of PD‐L1 ligands bound to PD‐1 expressed on TIL and TAM.^54^ Yi et al. PTRF was found to be associated with immunosuppression through analysis in public databases. Overexpression of PTRF significantly increased the level of PD‐L1 in GBM primary cell lines, and PTRF interacted with NEAT1 and stabilized its mRNA, and further inhibited UBXN1 expression to promote NF‐kB activity and thus enhanced PD‐L1 transcription. Moreover, PTRF could promote immune evasion in GBM cells by regulating PD‐1 binding and PD‐L1‐mediated T‐cell toxicity.^55^ It promotes immune evasion of GBM cells.^56^ Hoppmann et al. Glioblastoma proliferating cells (GPC) were treated in seven consecutive cell passages using 2.5 Gy of radiation to select GPC with increased colony‐forming properties and intrinsic or radiation‐induced resistance (rsGPC). Quantitative proteomic analysis revealed downregulation of MHC class I antigen processing and presentation mechanisms in cells with detergent‐resistant membranes (lipid rafts) in GPC vs. rsGPC. In addition, radio‐selected GPC showed reduced sensitivity to cytotoxic CD8+ T cell‐mediated killing. In conclusion, clinically relevant sublethal‐graded radiation leads to reduced expression of components of the MHC class I antigen processing and presentation pathway, resulting in immune escape.^57^ Upregulation of MHC‐I in glioma cell exosomes restores cellular antigen presentation and activates CD8+ T cells, thereby combining with immune checkpoint blockade to exert a potent antitumor immune response.^58^

**TABLE 1:** The potential role of tumor‐infiltrating T lymphocytes in the immune escape of glioma.

Molecular	Expression	Mechanism	Biological roles	Reference
miR‐21	Up	MiR‐21/PEG3	Promote cell growth, migration and invasion, accelerate	^45^
			cell apoptosis, and improve CD8+ T proliferation and cytotoxicity	
SDC1	Up	SDC1/CTSL/CD4	Promote the activation of CD4+ T cells and CD8+ T cells	^46^
INPP4B	Down	INPP4B/PI3K/AKT/PD‐L1	Inhibition of cell proliferation, migration, apoptosis resistance, PD‐L1 expression and T cell inhibition	^47^
PD‐1	Up	/	Reduce the proportion of CD8+ T cells and increase the Proportion of regulatory T cells	^48^
NTBR	Up	NTBR/TGF‐b/Treg	Promote cell proliferation and tumor immune escape	^93^
GARP	Up	/	Inhibit the function of e vector T cells	^49^
MHC‐I and APM components	Down	HDAC/MHC‐I‐APM/Wnt	Block CTL‐mediated killing effect	^50^
ICOSLG	Up	TNF‐α/NF‐κB/ICOSLG	Promote the expansion of T cells producing interleukin‐10	^51^
β‐ Catenin/TCF/LEF complex	Up	Wnt/EGFR/β‐catenin/TCF/LEF/CD274/PD‐L1	Inhibit the activation and infiltration of CD8+ T cells and promotes tumor growth	^52^
TLX	Up	TLX/PD‐L1	Increase the number of macrophages and cytotoxic lymphocyte infiltration and inhibit tumor growth	^53^
PTRF	Up	PTRF/NEAT1/UBXN1/NF‐kB/PD‐L1	Modulate T‐cell toxicity	^55^
Exosomal MHC‐I	Down	/	Restore antigen presentation of cells and activate CD8+ T cells	^57^

### 3.2 Tumor‐associated macrophages and immune escape

Monocytes are recruited by chemokines, cytokines, and growth factors generated by tumor cells or mesenchymal cells and further differentiated to produce tumor‐associated macrophage.^59^ TAM has long been classified as a type of immune cell with antitumor effects. Macrophages are derived from myeloid cell lines, yolk sacs, embryonic precursors of fetal liver progenitor cells or monocyte precursors of hematopoietic origin and have a good proliferative capacity.^60^ Macrophages can be classified into two main categories. They can differentiate into classically activated macrophages (M1 phenotype) upon stimulation by IFN‐γ, LPS, TNF‐α, etc.^61^ M1 macrophages produce inflammatory and immunostimulatory cytokines, trigger adaptive responses, secrete reactive oxygen species (ROS) and nitrogen intermediates, participate in host innate defense, and have a transformed cell with cytotoxic effects, mainly involved in the Th1‐type immune response against pathogen invasion and surveillance of tumor lesions.^62^ In addition, differentiation into alternative activated macrophages (M2 phenotype) is induced by interleukins (IL‐4, IL‐10, and IL‐13) secreted by helper T cells 2. The M2 phenotype is essential for immune responses mediated by helper T cells 2 involving humoral immunity, wound healing and tissue remodeling.^63^ Besides, the M2 phenotype produces growth factors that activate tissue repair and angiogenesis, has high clearance activity, and suppresses the adaptive immune response.^64^ The M2 phenotype can be subdivided into three subgroups, M2a, M2b, and M2c, with M2a macrophages triggered by IL‐4 or IL‐13 and M2b macrophages triggered by the immune complex toll‐like receptor (TLR) and M2c macrophages. TLR and IL‐1Ra, and M2c macrophages are stimulated by IL‐10, transforming growth factor‐β (TGF‐β), and glucocorticoids.^65^ Based on extensive studies, it is proposed that TAM in TME is mostly polarized to anti‐inflammatory macrophages (M2 phenotype), which have the potential to elevate the activity of IL‐10, TGF‐β, and hyperarginase‐1, and stimulate the expression of cell surface markers, in addition to promoting tumor angiogenesis, growth, and expression of various immunosuppressive cytokines.^66^ This is in contrast to most pro‐inflammatory mediators secreted by M1 macrophages, including TNF‐α, IL‐1b, and IL‐12.^67^

Tumor‐associated macrophages also have a vital role in immune escape in gliomas (Table 2). Mechanistic findings suggest that overexpression of MET upregulates PD‐L1 and phosphorylates STAT4, and activation of the MET/STAT4/PD‐L1 pathway and upregulation of macrophages predicts poor prognosis in primary GBM patients. The MET‐STAT4‐PD‐L1 regulatory axis and TAMs may contribute to tumor malignancy progression by promoting glioma immune escape.^68^ It was shown that the expression levels of TLX and PD‐L1 were significantly and positively correlated with the macrophage‐mediated immunosuppressive phenotype in glioma, while the expression levels of TLX were also positively correlated with PD‐L1 expression. Inhibition of TLX expression significantly inhibited tumor growth and promoted antitumor immune responses by decreasing PD‐L1 expression, glioma‐associated macrophage numbers, and increasing cytotoxic lymphocyte infiltration. Mechanistic findings suggest that TLX directly binds to the promoter region of PD‐L1 and activates PD‐L1 transcription. In conclusion, TLX leads to malignant progression and immunosuppression of glioma through transcriptional activation of PD‐L1 ligands bound to PD‐1 expressed on TIL and TAM.^54^ TMEM198B is highly expressed in glioma tissues and cell lines and mediates trimethylation on H3K4me3 by binding to the SET structural domain of SETD1B, thereby promoting the expression of PLAGL2. Moreover, TMEM198B is enriched in glioma‐derived exosomes (GDEs) and can be delivered to macrophages and enhance lipid accumulation and fatty acid oxidation (FAO), further inducing macrophages to polarize toward M2, thereby promoting immune escape of glioma cells.^69^

**TABLE 2:** The potential role of tumor‐associated macrophages in the immune escape of glioma.

Molecular	Expression	Mechanism	Biological roles	Reference
MET	Up	MET/STAT4/PD‐L1	Promote chemotaxis of macrophages	^67^
TLX	Up	TLX/PD‐1	Increase the number of glioma‐associated macrophages and Inhibit cytotoxic lymphocyte infiltration	^53^
TMEM198B	Up	TMEM198B/SETD1B/PLAGL2/ACLY/ELOVL6	Enhance lipid accumulation and fatty acid oxidation and induce Macrophages to polarize toward M2	^68^

### 3.3 Natural killer cells (NK) and immune escape

**FIGURE 5:**
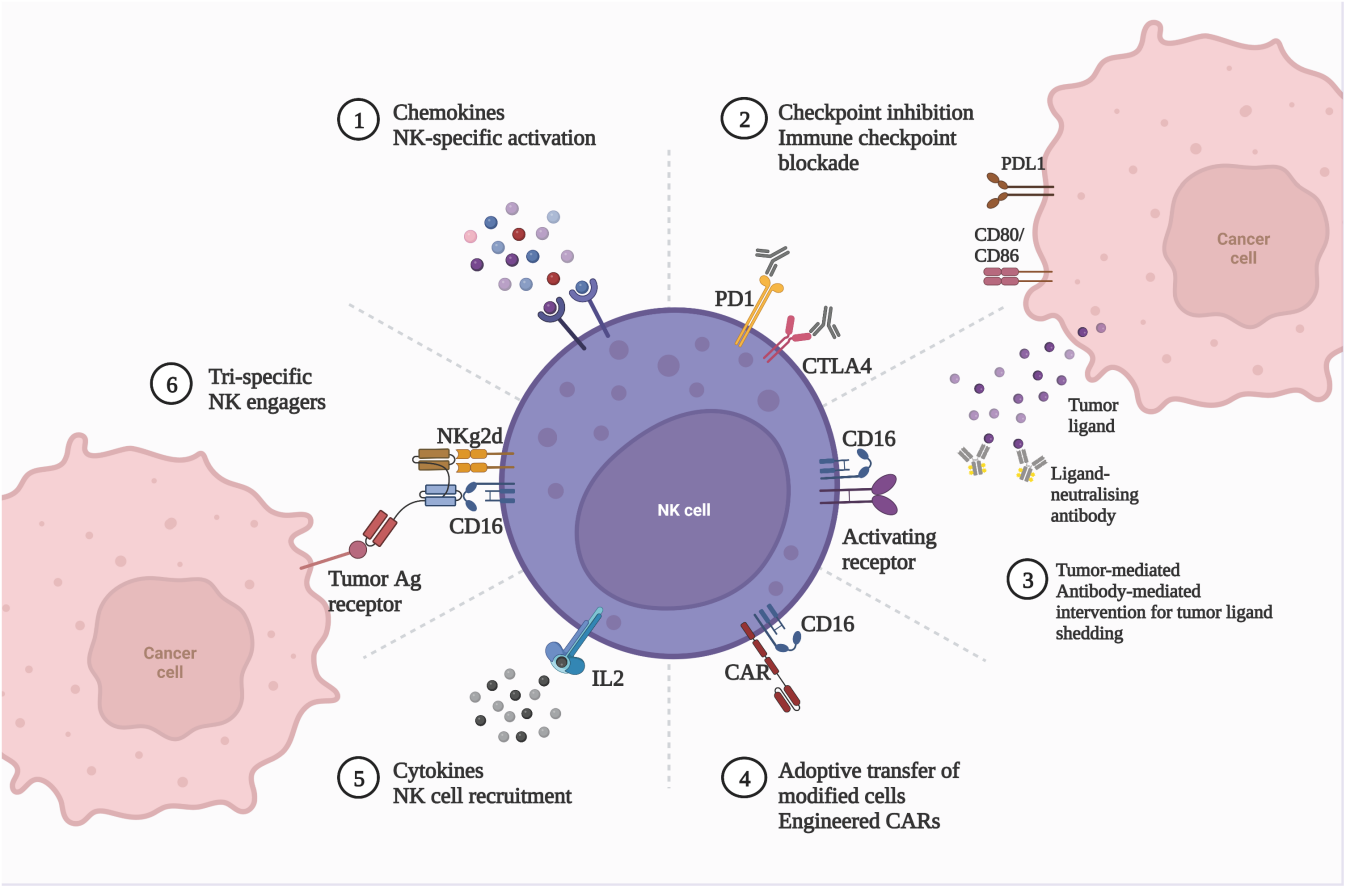
Approaches for NK Cell Immunotherapy. At present, there are many methods to use NK cells for immunotherapy, including chemokines NK specific activation, checkpoint inhibition and immune checkpoint blockade, tumor‐mediated and antibody‐mediated intervention for tumor light shielding, adaptive transfer of modified cells engineered CARs, cytotoxic NK cell reception and Tri specific NK enablers. Created with BioRender.com.

Natural killer (NK) cells are a significant component of the body's immune system and a major subset of lymphocytes that secrete perforin, interferon‐gamma (IFN‐γ), tumor necrosis factor (TNF), granulocyte‐macrophage stimulating factor (GM‐CSF), and macrophage inflammatory protein‐1 (MIP‐1) to enhance cytotoxicity during immune response.^70^ NK cells are not only associated with antimalignancy, antiviral infection, and immune regulation but also have a role in some of the most important immune functions.^71^ NK cells are not only associated with antimalignancy, antiviral infection, and immune regulation, but in some cases are related to hypersensitivity reactions and autoimmune diseases, recognizing target cells can also activate and promote the killing effect of killing mediators.^72^ Under physiological conditions, the normal function of NK cells is regulated and conditioned by both activating and inhibiting signals.^73^ However, some tumor cells can cause changes in NK cell function through different regulatory mechanisms that prevent them from performing their normal immunosurveillance function, resulting in tumor cells evading immunosurveillance.^74^ The approaches for NK Cell Immunotherapy were displayed in Figure 5.

Natural killer also has an important role in immune escape from glioma (Table 3). The expression of NKG2D ligands was significantly lower in IDH mutant glioma stem‐like cell lines than in IDH wild‐type cells. In addition, both IDH mutant glioma cells and astrocytes were resistant to NK cell‐mediated lysis, whereas decitabine increased NKG2D ligand expression and restored NK‐mediated lysis in IDH mutant cells in an NKG2D‐dependent manner. Mechanistic findings suggest that IDH mutant glioma cells can acquire resistance to NK cells by inhibiting ULBP1 and ULBP3 expression through NKG2D ligands, and decitabine‐mediated hypomethylation restores ULBP1 and ULBP3 expression in IDH mutant glioma cells, which may be a useful target for immune monitoring in patients with IDH mutant diffuse glioma.^75^ EZH2‐92aa, a protein encoded by cyclic EZH2, is significantly expressed in GBM and induces immune escape of GSC from NK cells. Mechanistic studies have shown that EZH2‐92aa is positively regulated by DDX3 and directly binds to the major histocompatibility complex class I peptide‐related sequence A/B (MICA/B) promoter to repress its transcription. In addition, EZH2‐92aa can indirectly repress ULBP transcription by stabilizing EZH2. Furthermore, inhibition of EZH2‐92aa expression significantly enhanced NK cell‐mediated GSC eradication in vitro and in vivo, and synergized with anti‐PD1 treatment. In conclusion, the above results show that EZH2‐92aa can produce tumor immunosuppressive function by regulating NK cell response and thus tumor immunosuppression.^76^

**TABLE 3:** The potential role of natural killer cells in the immune escape of glioma.

Molecular	Expression	Mechanism	Biological roles	Reference
NKG2D ligand	Down	NKG2D ligand/ULBP1和ULBP3	Restore NK‐mediated IDH mutant cell lysis	^74^
EZH2‐92aa	Up	DDX3/EZH2‐92aa/MICA/B	Induction of GSC immune escape from NK cells	^75^
		EZH2‐92aa/EZH2/ULBP		

### 3.4 Dendritic cells and immune escape

As antigen‐presenting cells, DCs are currently attracting increasing attention in tumor bio immunotherapy. DCs are the most powerful antigen‐presenting cells in the body and can directly activate Th and CTL in the immune system, as well as cause B cells to produce antibodies.^77^ Tumor antigen‐sensitized DCs in vitro were obtained by isolation and purification of DCs, which can lead to T cell activation through high levels of stimulation of MHC class I and MHC class II molecules.^78^ Also, DCs can induce the secretion of IFN‐α, IFN‐γ, IL‐3, IL‐12, and other factors to produce relevant immune responses.^79^ In addition, DCs can be genetically engineered to enhance the antitumor immune response.^80^

Dendritic cells also have an important role in the immune escape of gliomas (Table 4). Macrophage MIF expression is increased in glioblastoma and promotes malignant progression of the tumor as well as evasion of immune surveillance. Recombinant human MIF (rhMIF) activates the RhoA‐ROCK1 pathway and promotes the formation of F‐actin fibers, thereby increasing autophagy in glioblastoma cells. In addition, exogenous rhMIF significantly inhibited the migration of immature DCs (iDC) and mature DCs (mDC). In conclusion, MIF can lead to malignant progression of GBM by inducing autophagy and evasion of DC monitoring in glioblastoma.^81^ Also, found that GSCs can maintain an immunosuppressive microenvironment by reducing the peripheral extracellular ATP concentration through upregulation of CD39 expression by SOX2 binding to the CD39 promoter. In addition, adriamycin (ADM) promoted the release of ATP and recruited DCs to phagocytose GSCs, and ADM treatment and DC phagocytosis suppressed CD39 expression and further increased the extracellular ATP concentration. Results from animal studies showed that the combination of ADM and CD39 blockers increased immune cell infiltration and reduced tumor size.^82^ It was shown that stimulation with tumor‐conditioned medium (TCM) prepared from glioma cells inhibited the induction of DC maturation by suppressing the expression of CD80, CD86, and IL‐12 p70, and promoting IL‐10 expression. In addition, TCM exposure promoted Nrf2 expression and transcriptional activity in DCs and could further promote T cell proliferation as well as display a Th1 response through IFN‐γ production, enhancing the cytotoxic capacity of T cells against glioma cells.^83^ Membrane‐linked protein‐1 (ANXA1) expression is significantly increased in GBM and predicts a poor prognosis. Inhibition of ANXA1 inhibits proliferation, migration, and invasion of GBM cells and enhances their radiosensitivity. Besides, ANXA1 is also involved in DC maturation and may inhibit DC infiltration. Mechanistic findings suggest that ANXA1 significantly promotes IL‐8 production and p65 phosphorylation levels and thus mediates tumor immune escape.^84^

**TABLE 4:** The potential role of dendritic cells in the immune escape of glioma.

Molecular	Expression	Mechanism	Biological roles	Reference
rhMIF	Up	NKG2D ligand/ULBP1/ULBP3	Promote the formation of F‐Actin fiber, increase cell autophagy, and inhibit	^80^
			The transformation from immature DC to mature DC	
Adriamycin	/	ADM/CD39/ATP	Inhibits immune cell infiltration and promotes tumor growth	^81^
Tumor conditioned medium	/	TCM/IL‐10	Inhibition of induced maturation of DC	^82^
		TCM/Nrf2/IFN‐γ	Promote T cell proliferation and cytotoxicity	
ANXA1	Up	ANXA1/IL‐8/p‐p65	Promote cell proliferation, migration and invasion, inhibit tumor infiltration	^83^

### 3.5 Other mechanisms involved in immune escape in the TME of glioma

HOTAIR is significantly expressed in glioma tissues and activates the expression of NF‐kB, TNFa, MAPK, and other inflammatory signaling pathway‐related proteins to activate the immune response, T cell costimulation, and RNA polymerase II to initiate transcription in glioma. Mechanistic studies have shown that HOTAIR can promote NF‐kB phosphorylation and nuclear translocation and IkBa phosphorylation by inhibiting the expression of UBXN1, a protein upstream of NF‐kB. In addition, inhibition of HOTAIR expression could inhibit PD‐L1 protein expression. In conclusion, HOTAIR can drive aberrant gene transcription and immune escape in tumor cells by regulating the NF‐kB signaling axis.^85^ Phosphorylated signal transducer and activator of transcription 1 and mycovirus resistance protein A (MxA) can be constitutively expressed in the lack of exogenous IFN‐β. MxA expression is increased in glioma tissues, while type I interferon induces intracellular signaling through the IFNAR1/2 axis. Inhibition of IFNAR1 or IFNAR2 expression significantly suppressed PD‐L1 and MHC class I and II expression and enhanced the sensitivity of natural killer immune cell lysis. The above results suggest that autocrine IFN signaling promotes immune evasion of glioma cells.^86^ Ma et al. showed that the combined transcriptional profiling of Oct4/Sox2 co‐expressing GSC and differentiated GBM cells identified a microenvironment consisting of an immunosuppressive transcriptome composed of multiple immunosuppressive checkpoints (i.e., PD‐L1, CD70, A2aR, and TDO) synergistically induced by Oct4 and Sox2 and dysregulation of immunosuppressive tumor‐associated cytokines and chemokines. Mechanistic findings suggest a role for the induction and function of BRD/H3k27Ac‐dependent immunosuppressive genes in the immunosuppressive phenotype of GSC. In conclusion, the reprogramming transcription factors (TFs) Oct4 and Sox2 can contribute to the formation of the GBM immunosuppressive microenvironment by driving the GSC immunomodulatory transcriptome and thus the GBM immunosuppressive microenvironment.^87^ It was shown that the expression levels of TLX and PD‐L1 were significantly and positively correlated with the macrophage‐mediated immunosuppressive phenotype in glioma, while the expression levels of TLX were also positively correlated with PD‐L1 expression. Inhibition of TLX expression significantly inhibited tumor growth and promoted antitumor immune responses by decreasing PD‐L1 expression, glioma‐associated macrophage numbers, and increasing cytotoxic lymphocyte infiltration. Mechanistic findings suggest that TLX directly binds to the promoter region of PD‐L1 and activates PD‐L1 transcription. In conclusion, TLX leads to malignant progression and immunosuppression of gliomas through transcriptional activation of PD‐L1 ligands bound to PD‐1 expressed on TIL and TAM.^54^ INPP4B expression is significantly reduced in gliomas, and overexpression of INPP4B inhibits glioma cell proliferation, migration, apoptosis resistance, PD‐L1 expression, and T‐cell suppression. Mechanistic findings showed that INPP4B inhibited PD‐L1 expression and thus glioma immune escape through downregulation of PI3K/AKT signaling.^47^

## 4 FUTURE PROSPECTS AND DISCUSSION

**FIGURE 6:**
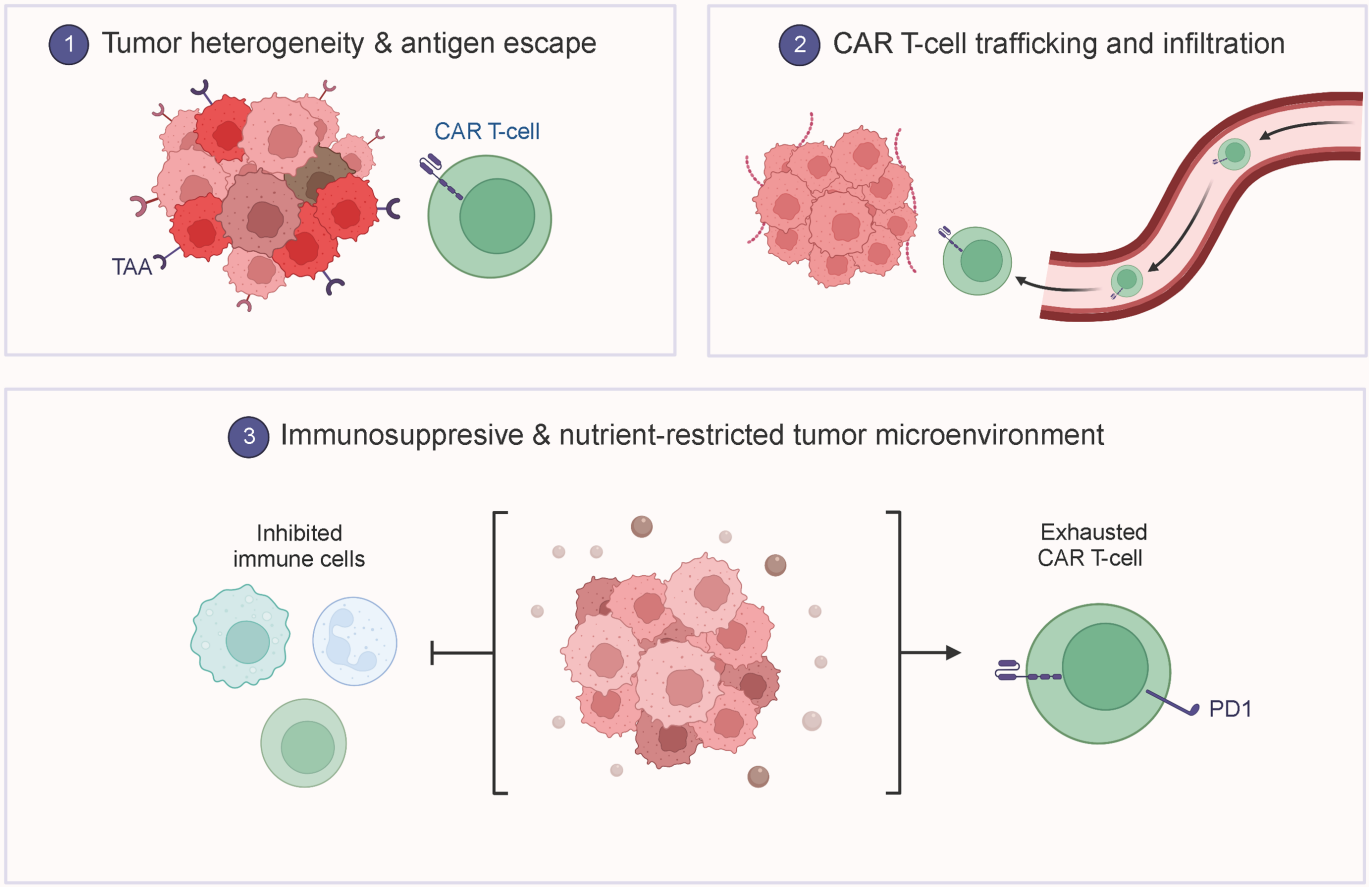
The limitations of CAR‐T therapy. The limitations of CAR‐T therapy include tumor heterogeneity, antigen escape, CAR‐T‐cell trafficking and infiltration, immunosuppressive and nutrient‐restricted tumor microenvironment. Created with BioRender.com.

The fundamental of tumor treatment is the capability of the body's immune system to recognize and distinguish normal cells from diseased cells, and to specifically recognize, kill, and remove tumor cells by recognizing antigens on the surface of tumors.^88^ In addition to surgical treatment, radiotherapy, and chemotherapy, there is also a growing interest in immunotherapy, including PD‐L1/PDL2‐PD‐1, and related signaling pathways, CTLA‐4 inhibitors and CAR‐T therapy.^89^, ^90^ However, CAR‐T treatment still has many shortcomings. The limitations of CAR‐T therapy were showed in Figure 6. Immunotherapy is currently one of the most attractive therapeutic strategies for cancer patients. An increasing number of recognized immune checkpoint inhibitors (including PD‐1 and CTLA4 inhibitors) affect the local tumor immune environment by blocking the PD‐1/PD‐L1 and CTLA4 signaling pathways and have been used to treat certain types of cancer.^91^, ^92^ The phenomenon that tumor cells use all kinds of mechanisms to escape the surveillance and attack of the body's immune system so that they can continue to grow and metastasize is called tumor cell immune escape, which is a crucial strategy for tumor survival and progression.^93^ In TME, interactions between glioma cells and other immune cells are responsible for promoting immune escape of glioma cells. Although glioma cells can influence the differentiation and function of immune cells, further studies are needed to determine whether the intrinsic mechanisms by which different molecules and targets affect immune cells differ in different types of gliomas. Both tumor genetics and immune phenotypes can be used as predictive and prognostic biomarkers for glioma. In the future, we should further investigate the local and systemic antitumor immune characteristics, combine genomic data and immune phenotypes, and make full use of the power of genetics, immunology, and translational medicine to design effective treatment plans for surgery, radiotherapy, antiangiogenic targeted therapy, immunotherapy, etc., with individualized differences to improve the overall survival of glioma patients. The goal is to increase the OS rate of glioma patients.

In summary, immunotherapy has improved the treatment of many patients with previously poor prognosis of solid tumors but has not shown significant efficacy in the treatment of glioma. In this paper, we reviewed the research on immune response mechanism and immune escape mechanism of tumor cells in glioma, with the aim of providing reference for the subsequent therapeutic research.

## CONFLICT OF INTEREST STATEMENT

We declare that we have no financial or personal relationships that could inappropriately influence or bias the content of this article. Additionally, we have no conflicts of interest, including but not limited to commercial, personal, political, intellectual, or religious interests, that could be perceived as influencing this work. This manuscript represents original research and has not been previously published, nor is it under consideration for publication elsewhere.

## Data Availability

Data sharing is not applicable to this article as no new data were created or analyzed in this study.
